# Supplementary material for: Physical, mental and social status after COVID-19 recovery in Nepal: A mixed method study
Source: PLoS One. 2023 Sep 5;18(9):e0290693. doi: 10.1371/journal.pone.0290693 (PMC10479937; doi:10.1371/journal.pone.0290693)
Supplement: S1 Questionnaire — (DOCX) [file pone.0290693.s001.docx]

# Annex II: Questionnaire

**Assessment of Post-Recovery Impact on Patients with COVID-19 in Nepal**

**Socio-demographic information:**

**Registration number**: Full

Name:

**Address at the time of Diagnosis:**

Province: District: GP/MP

Ward

No: Tole: Village

**Address:**

**Permanent**

Province: District: GP/MP Ward No: Tole: Village

| **SN** | **Question** | **Answer** | **Skip** |
| --- | --- | --- | --- |
| 1 | Gender | 1. Male  2. Female  3. Not specified/Unknown |  |
| 2 | Age | [ ][ ]years [ ][ ]months |  |
| 3 | Marital status | 1. Unmarried  2. Married  3. Divorced /Separated  4. Single |  |

| 4 | Ethnicity | 1. Hill Brahminn  2. Hill Chhetri  3. Terai Brahmin/ Chhetri  4. Hill Dalit  5. Terai Dalit  6. Newar  7. Other hill janajati  8. Terai janajati |  |
| --- | --- | --- | --- |
|  |  | 9. Other terai caste  10. Muslim  88. Others  99. Unknown |  |
| 5 | Religion | 1. Hindu  2. Buddhist  3. Muslim  4. Christian  5. Kirat  88. Other (Specify) |  |
| 6 | Education | 1. Illiterate  2. Literate but no formal schooling  3. Primary education (1to7)  4. Secondary education (8to10)  5. Higher  6. Not Applicable |  |
| 7 | Occupation | 1. Employed as a Healthcare Worker 2.  Employed in a microbiology laboratory  3. Employed in other profession |  |
| 8 | Travelling history | 1. Abroad  2. Inter state  3. Inter district  4. Inter GP/MP  5. not travelled |  |
| 9 | Where do you treat yourself during  COVID-19 tenure | 1. Self-isolation  2. Hospital discharge  3. Quarantine |  |
| 10 | Name of treating hospital | ……………………………. |  |
| 11 | Referral (If any) | ……………………… |  |
| 12 | Admitted date |  |  |
| 13 | Discharged date |  |  |
| **COVID-19 recovered patient characteristics** | | | |

| 13 | Diabetes | 1. Yes  2. No | | | |  |
| --- | --- | --- | --- | --- | --- | --- |
| 14 | Heart disease | 1. Yes  2. No | | | |  |
| 15 | Chronic lung disease | 1. Yes  2. No | | | |  |
| 16 | Chronic liver disease | 1. Yes  2. No | | | |  |
| 17 | Active tuberculosis | 1. Yes  2. No | | | |  |
| 18 | Asthma | 1. Yes  2. No | | | |  |
| 19 | HIV infection | 1. Yes  2. No | | | |  |
|  |  | **Before infected**  **from COVID-19** | | **After infected**  **from COVID-19** | |  |
|  |  | **Yes (1)** | **No(2)** | **Yes(1)** | **No(2)** |  |
| 20 | Smoker |  |  |  |  |  |
| 21 | Alcohol consumption |  |  |  |  |  |
| **Prese nt health conditions** | | | | | | |
| 23 | How is your current health condition | Normal……………………………..1  Few problems………………………2  Complicated………………………..3 | | | |  |
| 24 | How is your current weight | High………………………………..1  Low………………………………..2  Normal……………………………..3 | | | |  |

**Physical well-being**: **In the past 4 weeks, did the patient have any difficulty or need help with:**

| **S.N** | **Questions** | **Normal(1)** | **Has**  **difficulty, but does by self(2)** | **Requires assistance(3)** | **Dependent(4)** |
| --- | --- | --- | --- | --- | --- |
| 1. | Doing household chores  (cooking food, washing clothes, cleaning and doing any other household work) |  |  |  |  |
| 2. | Activities outside home  (agricultural work, animal husbandry, buying household goods etc.) |  |  |  |  |
| 3. | Activities related to  financial income (business affairs, working in office or daily wages) |  |  |  |  |

**Social well being**

| **S.N** | **Questions** | **Fully agree (1)** | **Agree(2)** | **Disagree(3)** | **Fully**  **Disagree(4)** |
| --- | --- | --- | --- | --- | --- |
| 1. | There are people I can depend  on to help me if I really need it. |  |  |  |  |
| 2. | There are people who join the  same social activities I do. |  |  |  |  |
| 3. | I have close relationships in  society that provide me with a sense of emotional security and well-being. |  |  |  |  |
| 4. | There is someone I could talk  to about important decisions in my life. |  |  |  |  |
| 5. | I have relationships in  workplace where my competence and skills are recognized. |  |  |  |  |
| 6. | I am able to be a part of a  group of people who share my attitudes and beliefs. |  |  |  |  |
| 7. | I feel a strong emotional bond  with at least one other person. |  |  |  |  |

**Mental Well-being**

| **S.N** | **Questions** | **No (1)** | **Some (2)** | **High(3)** |
| --- | --- | --- | --- | --- |
| 1. | Repeated disturbing and unwanted  thoughts about the COVID-19 outbreak |  |  |  |
| 2. | Trying to avoid information or  reminders about the COVID-19 outbreak |  |  |  |
| 3. | Have you had any negative?  feelings/thoughts towards yourself and others? (Probe: I am a bad person, I cannot trust anyone, society is not secure to live) |  |  |  |
| 4. | Work/business Stress |  |  |  |
| 5. | General stress at home |  |  |  |
| 6. | Severe financial stress/Due to  unemployment |  |  |  |

# Annex III: Nepali Questionnaire

**g]kfndf COVID-19/sf]/f]gf efO/; af6 ;+qmldt eO{ lgsf] ePsf la/fdLx¿df k/]sf] k|efjsf] d"Nof+sg**

**;fdflhs / hg;f+lVosLo ljj/0f**

Registration number………………………………

k"/f gfd========================================================

:yfoL 7]ufgf

k|b]z ………………. lhNnf ...............................................

ufpFkflnsf÷ gu/kflnsf............................................. j8f g+ ...............................

6f]n................................ .............

c:yfO{ 7]ufgf

k|b]z ………………. lhNnf.....................................

ufpFkflnsf÷ gu/kflnsf............................................. j8f g+ ...............................

6f]n................................ .............

| qm ;+ | k\|:gfjfnL | pQ/ | :sLk |
| --- | --- | --- | --- |
| ! | lnË | Dflxnf=============================================================! k"¿if==============================================================@ gtflsPsf] ÷yfxf gePsf] ==================================# |  |
| @ | pd]/ | ===============================jif{ |  |
| # | j}jflxs l:ylt | ljjflxt =========================================================! cljjflxt ========================================================@ 5'l§Psf] ===========================================================#  Psn================================================================$ |  |
| $ | hftL | Afx'g=============================================================! IfqL=================================================================@ blnt ==============================================================# g]jf/ ===============================================================$ hghftL============================================================% d'l:nd ============================================================^ cGo t/fO{ hftL =================================================& |  |
| % | wd{ | lxGb'================================================================! a'l4i6 =============================================================@ d'lind =============================================================# OzfO{ ==============================================================$ cGo eP v'nfpg]===============================================** |  |
| ^ | z}lIfs :t/ | clzlIft===========================================================! ;fIf/ t/ cf}krfl/s lzIff gePsf]========================@ k\|fylds lzIff -! b]lv &_=======================================# dfWolds lzIff -*b]lv !)_=====================================$ pRr lzIff===========================================================% cGo- v'nfpg]_ ==================================================** |  |
| & | k]zf | :jf:YosdL{sf] ¿kdf sfo{/t=================================! dfOqmf]afof]nfhL k\|of]uzfnfdf sfo{/t]=====================@  cGo k]zfdf sfo{/t===============================================# |  |
| * | ofqf u/]sf] 7fpF | ljb]z===================================================================! cGt/ k\|b]z ==========================================================@  cGt/ lhNnf========================================================#  cGt/ gu/kflnsf jf ufpkflnsf============================$  ofqf gu/]sf]=========================================================% |  |
| ( | tkfO{ sf]/f]gf af6 ;+qmldt eO{ lgsf] x'g] cjlw e/ tkfO{n] sxfF a;]/ cfˆgf] pkrf/ u/fO{ lgsf] x'g'eof] | 3/df cfO;f]n];gdf -cnu_ a;]/ ==========================!  c:ktfndf==========================================================@  sjf/]lG6gdf=========================================================#  cGo -v'nfpg]_======================================================$ |  |

pkrf/ ul/Psf] c:ktfn==================================================================

l/km/ ul/Psf] c:ktfn -obL ePdf_=================================================================================== egf{ ldlt==================================================================8L;rfh{ ldlt================================================

**sf]/f]gf efO/;sf] ;+qmd0faf6 lgsf] ePsf JolQdf :jf:Yo ljj/0f**

| **qm ;+** | **k\|:gfjnL** |  | | **pQ/** |  | | **:sLk** |
| --- | --- | --- | --- | --- | --- | --- | --- |
|  |  | **5 (!)** | |  | **5}g (@)** | |  |
| ! | dw'd]x |  | |  |  | |  |
| @ | d'6' /f]u |  | |  |  | |  |
| # | hl6n kmf]S;f]sf] /f]u |  | |  |  | |  |
| $ | hl6n sn]hf]sf] /f]u |  | |  |  | |  |
| % | ;lqmo Ifo/f]u |  | |  |  | |  |
| ^ | bd |  | |  |  | |  |
| & | PrcfO{eL ;+qmd0f |  | |  |  | |  |
| * | cGo |  | |  |  | |  |
|  |  |  | |  |  | |  |
|  | **k\|:gfjnL** | **sf]/f]gf efO/;sf] ;+qmd0f cl3** | | | **sf/f]gf efO/;sf] ;+qmd0f ] kl5** | |  |
|  |  | **u5'{ (!)** | **ub}{g (@)** | | **u5'{ (!)** | **ub}{g (@)** |  |
| ( | w'd\|kfg ;]jg |  |  | |  |  |  |
| !) | dBkfg ;]jg |  |  | |  |  |  |

**sf]/f]gf efO/;sf] ;+qmldtsf jt{dfg :jf:Yo cj:yf**

| **qm ;+** | **k\|:gfjnL** | **pQ/** | **:sLk** |
| --- | --- | --- | --- |
| ! | tkfOsf] jt{dfg :jf:Yo cj:yf s:tf] 5 | /fd\|f]=============================================================! ;fdfGo ;d:of==============================================@ uDeL/ ;d:of================================================# |  |
| @ | tkfOsf] xfn tf}n s:tf] 5 | pRr============================================================! sd=============================================================@ l7s}=============================================================# |  |

\

**For physical-well being**

sfof{Tds ultlalwaf/] k|:gfjln **(Functional Activities Questionnaires)**

laut $ xKtfdf tn pNn]lvt lqmofsnfk ug{ la/fdLnfO{ s]lx c;xh jf d2t cfj:os k/]sf] lyof]<

| qm ;+ | k\|:gfjfnL | ;fdfGo  **(**!**)** | sl7gfO{ 5 t/  cfkm}+n] u5{' **(**@**)** | ;xof]u cfjZos 5**(**#**)** | lge{/ **(**$**)** |
| --- | --- | --- | --- | --- | --- |
| ! | 3/ leqsf] sfdsfh ug{  - vfgf ksfpg, n'uf w'g, 3/ ;kmf ug{ jf cGo 3/fo;L sfdsfh ug{_ |  |  |  |  |
| @ | 3/ aflx/sf] sfdsfh ug{- v]ltkftL, ufO{ j:t' sf] x]/rfx, 3/df rflxg] cfj:os j:t' lsGg jf cGo rfx]sf] sfd ug{_ |  |  |  |  |
| # | cfly{s cfocfh{g sf] lqmofsnfk -Jofkf/ Joj;fo, sfof{nodf sfd ug{, b}lgs cfo cfh{gsf] sfdsfh ug{_. |  |  |  |  |

**Social well-being (**cfˆgf] ;dfhdf sf/f]gf ;+qmldt ePsf] yfxf geP of] v08 ] -;]Szg_ 5f]8\g]**)**

| qm ;+ | k\|:gfjfnL | k"0f{  ;xdt  **(**!**)** | ;xdt**(**@**)** | c;xdt  **(**#**)** | k"0f{  c;xdt  **(**$**)** |
| --- | --- | --- | --- | --- | --- |
| ! | dnfO{ cfjZos k/]sf] a]nfdf dnfO{ ;xof]u ug d]/f ;fyL]{ x? jf cGo JolQmx? x'g' x'G5 |  |  |  |  |
| @ | d ;dfj]z ePsf] ;fdflhs sfddf -v]ns'b, ;/;kmfO{, cflb_ ;dfhsf] cGo JolQmx? klg  ;fd]n x'g] u/]sf 5g |  |  |  |  |
| # | d]/f] cfkm" a:g] ;dfh;+u 3lgi6 ;DaGw 5 h:n] ubf{ d ;fdlhs / efjfgfTdfs ;'/Iff sf] cg'e"lt u5{' |  |  |  |  |
| $ | d]/f ;fy df To:tf JolQm x? 5g \ hf] ;+u d d]/f] cfˆgf] lhGbuL sf] dxTjk"0f{ lg0f{ox?sf] af/] s'/f ug{ ;Sb5' |  |  |  |  |
| % | d sfd ug]{ 7fpF df d]/f] nufe 5 h:n] ubf{ d]/f] sfdsf] dfGotf / d'Nof+sg x'G5 |  |  |  |  |
| ^ | d ;dfhsf] Pp6f lx:;f aGg ;kmn ePsf]  5' h'g sf/0f d]/f] dgf]j[lQ / ljZjf; a9]sf]  5 |  |  |  |  |
| & | d]/f] ;fy df slDt df Pshgf o:tf JolQm 5g \ hf] ;+u d]/f] alnof] efjgfTds ;DaGw /x]sf] 5 |  |  |  |  |

**Mental well-being**

| qm ;+ | k\|:gfjfnL | 5}g**(**!**)** | s]lx ePsf] 5  **(**@**)** | w]/} ePsf] 5 **(**#**)** |
| --- | --- | --- | --- | --- |
| ! | s] tkfO{n] sf]/f]gf efO{/; ;+qmd0f s'g} t/Lsfn] km]/L dx;'; ug{'ePsf] 5 < h:t}M To;sf] af/]df ;kgf b]Vg] jf s8f -lal;{g} g;Sg]_ ofbx? cfpg' jf ;+qmd0f k"g cfˆgf] z/L/df cg'ej ug{' |  |  |  |
| @ | s] tkfO{n] sf]/f]gf efO{/; ;+qmd0fsf] af/]df g;f]Rg jf s'/f gug{ k\|of; ug{'eof] < |  |  |  |
| # | s] tkfO{nfO{ cfkm' jf c? k\|lt w]/} gsf/fTds wf/0ff /ljrf/ -h:t}M d g/fd\|f] dfG5] x'F,s;}nfO{ ljZjf; ug{  ;lsb}g,;+;f/ c;'/lIft 5 cflb\ _ cfPsf] 5 |  |  |  |
| $ | tkfO{ nfO{ cfˆgf] sfd jf Jofkf/df tgfj dx;"; ePsf] 5 |  |  |  |
| % | tkfO{ nfO{ cfˆgf] 3/df ;fdfGo tgfj dx;"; ePsf] 5 |  |  |  |
| ^ | tkfO{ nfO{ cfyL{s tgfj -a]/f]huf/L jf cGo sf/0f  _dx;"; ePsf] 5 (If the respondent is not a working population skip the question) |  |  |  |

# Annex IV: IDI guidelines

**g]kfndf COVID-19/sf]/f]gf efO/; af6 ;+qmldt eO{ lgsf] ePsf la/fdLx¿df k/]sf] k|efjsf] d"Nof+sg**

**;fdflhs / hg;f+lVosLo ljj/0f**

Registration number………………………………

k"/f gfd========================================================

**:yfoL 7]ufgf**

k|b]z ………………. lhNnf ...............................................

ufpFkflnsf÷ gu/kflnsf............................................. j8f g+ ...............................

6f]n................................ .............

**c:yfO{ 7]ufgf**

k|b]z ………………. lhNnf ...............................................

ufpFkflnsf÷ gu/kflnsf............................................. j8f g+ ...............................

6f]n................................ .............

lnË, pd]/, j}jflxs l:ylt, hftL, wd{, z}lIfs :t/, k]zf, ofqf u/]sf] 7fpF

sf]/f]gfaf6 ;+qmldt eO{ lgsf] x'g] a]nf ;Dd tkfO{n] sxfF a;]/ cfˆgf] pkrf/ u/fpg'eof] xfdLnfO{ atfO{lbg' ;Sg' x'G5 -k|f]aM 3/df cfO;f]n];gdf -cnu_ a;]/, c:ktfndf, sjf/]lG6gdf, kl/jf/;+u}

pkrf/ jf l/km/ ul/Psf] c:ktfn=============================================================================

**zf/Ll/s :jf:Yo (Physical health)**

1. Can you tell us about your current physical health condition? Current physical health issues (Probe) (Feeling tired or having little energy, Poor appetite or overeating, Fever, Dry cough, Sore throat, Diarrhea, Fatigue, Sore eyes, conjunctivitis, water eyes, Loss of smell, Respiratory infections, problem on heart, kidneys, and brain, Loss of taste, No symptoms, etc.)

!_ xfn tkfO{+sf] zf/Ll/s :jf:Yo cj:yf af/] atfO{ lbg'xf];\ . -k|f]a: ysfg dx;'; jf tfut sd ePsf] dx;';, ef]s sd nfUg] jf cTolws vfg dg nfUg], Hj/f], ;'Vvf vf]sL, 3fF6L b'Vg], kvfnf nfUg], cfFvfx¿ b'Vg], cfFvfaf6 kfgL aUg], uGw x/fpFb} hfg], Zjf;k|Zjf;df ;d:of, Åbo, lduf}{nf, / dl:tisdf ;d:of, vfg] s'/fsf] :jfb x/fpg], s'g} nIf0f gePsf], cflb_

2. What physical health problems /issues did you face when you were infected with COVID-19? (Probe) (Feeling tired or having little energy, Poor appetite or overeating, Fever, Dry cough, Sore throat, Diarrhea, Fatigue, Sore eyes, conjunctivitis, water eyes, Loss of smell, Respiratory infections, problem on heart, kidneys, and brain, Loss of taste, No symptoms, etc.)

@_ tkfO{+ sf]/f]gf efO/; af6 ;+qmldt x'Fbf tkfO{+nfO{ s] s:tf :jf:Yo ;d:of b]vf k/]sf] lyP < -k|f]a: ysfg dx;'; jf tfut sd ePsf] dx;';, ef]s sd nfUg] jf cTolws vfg dg nfUg], Hj/f], ;'Vvf vf]sL, 3fF6L b'Vg], kvfnf nfUg], ysfg, cfFvfx¿ b'Vg], cfFvfaf6 kfgL aUg], uGw x/fpFb} hfg], Zjf;k|0ffnL ;+qmd0f, Åbo, lduf}{nf, / dl:tisdf ;d:of, vfg] s'/fsf] :jfb x/fpg], s'g} nIf0f gePsf], cflb_

3. Tell us in detail about daily functional activities (Probe) (food consumption pattern (tea, turmeric, alcohol, onion prevents corona), physical activities, self-isolate, etc.)

#_ tkfO{+ cfkm'n] b}lgs ?kdf ul/g] sfd x? af/] lj:t[t ¿kdf atfO{ lbg ;Sg' x'G5< -k|f]a: vfgf vfg] z}nL -lrof, a];f/, /S;L, Kofh_ zf/Ll/s ultljlwx?, c?af6 cfkm" cnu a:g], cflb_

4. Do you have any more experiences to tell us about physical wellbeing?

$_ tkfO{;Fu zf/Ll/s :jf:Yosf] af/]df s'g} yk cg'ejx¿ eP xfdLnfO{ atfO{lbg'xf]; <

**dfgl;s :jf:Yo (Mental health (Psychological well-being))**

1. Have you felt any stress or anxiety or loneliness? Do you think it is due to COVID-19 only, or additionally, due to the overload of information you’re getting?

!_ s] tkfO{+n] s'g} lsl;dsf] tgfj, lrGtf jf PSnf]kg dx;'; ug{'ePsf] 5 < olb 5 eg], COVID-19 af6 ;+qmldt ePsf] sf/0f o:tf] dx;'; ePsf] xf] jf o:tf] dx;"; ug{'df cGo s'g} sf/0f 5g <

2. What measures have you taken to cope with the stress/anxiety/loneliness? (Probe) (Coping and self-care styles: speculation, isolation, distraction, self-consciousness, humor, rationalization)

@_ tgfj, lrGtf / PSnf]kgsf] ;fdgf ug{ tkfO{n] s]-s:tf pkfox¿ ckgfO{ /fVg' ePsf] 5 -k|f]a: ;fdgf ug]{ / cfˆgf] Wofg /fVg], cnu a:g], distraction, self-consciousness, humor, rationalization)

3. How has your psychological state of mind been in the past few days? (anxiety, depression, post-traumatic stress disorder (PTSD), etc.) What is your help seeking behaviors?

#_ ljut s]lx lbgdf sf]/f]gf efO/; af6 ;+qmldt ePsf] sf/0fn] tkfO{sf] dfgl;s cj:yfdf km/s kfpg" ePsf] 5 < tkfO{nfO{ cfj:os k/]sf] v08df sf] ;Fu ;xof]u lng] ug'{ ePsf] 5 .

**;fdflhs :jf:Yo (Social health/Social wellbeing)**

1. What is the social perception (Family, relatives, society) of COVID-19? (Probe) Experience, Risk perception, Community exposure Awareness)

!_ tkfO{ k|lt 3/kl/jf/, cfˆmGt / ;fdflhs wf/0ff s:tf] /x]sf] 5 . ;fy} s:tf] ?kdf lnPsf 5g\ -af]ln rfln, ;/;xof]u, cflb_ < -k|f]a: 3/kl/jf/ leq a'jf, cfdf, ;f;' ;;'/f, >Ldfg, >Ldlt, 5f]/f, 5f]/L, cflb cfˆmGt leq 3/kl/jf/ jf cfˆm';Fusf] gftfuf]tfsf dfG5] / ;fdflhs eGgfn] sfo{fno, :s'n, cflbdf e]l6g] dflg;x?_ cg'ej, hf]lvd x'g ;Sg] ;DalGw 1fg, ;fd'bflos ;+qmd0fsf] hfgsf/L

2. How do you spend your time after COVID-19 post-recovery? (Probe) (Daily functional activities and Socio-economic productivity, Coping and self-care styles)

@_ tkfO{n] cfˆgf] ;do s;/L latfO{/fVg' ePsf] 5 < hflu/, 3/fo;L sfd, cflb -k|f]a: b}lgs ultljlwx¿ / ;fdflhs, cfly{s pTkfbg, ;fdflhs e]befj, ;d:of ;fdgf ug]{ / cfˆgf] Wofg /fVg]_

s] tkfO{ KnfHdf (Plasma) bfg ug{ OR5's x'g'x'G5 <
